# Treatment of Chronic Haemophilic Synovitis with PRP: Clinical and *In Vitro* Studies

**DOI:** 10.3390/ijms25179140

**Published:** 2024-08-23

**Authors:** Horacio Caviglia, María Eulalia Landro, Paula Oneto, Guillermo Cambiaggi, Gustavo Galatro, Micaela Berni, Laura Caliogna, Eugenio Antonio Carrera Silva, Gianluigi Pasta

**Affiliations:** 1 Department of Medicine, Argentine Catholic University, Buenos Aires C1032ABS, Argentina; hacbagenoba@gmail.com (H.C.); mlandro@gmail.com (M.E.L.); 2 Haemophilia Foundation, Buenos Aires 3485, Argentina; guillecambiaggi@hotmail.com (G.C.); ggalatro@hotmail.com (G.G.); 3 Department of Traumatology, Juan A. Fernàndez Hospital, Buenos Aires 3356, Argentina; 4 Laboratory of Experimental Thrombosis, Institute of Experimental Medicine-CONICET, National Academy of Medicine, Buenos Aires C1032ABS, Argentina; onetopaula7@gmail.com (P.O.); carrerasilva@yahoo.com.ar (E.A.C.S.); 5 Department of Clinical, Surgical, Diagnostic and Pediatric Sciences, University of Pavia, 27100 Pavia, Italy; micaela.berni@hotmail.com; 6 Orthopedics and Traumatology Clinic, IRCCS Policlinico San Matteo Foundation, 27100 Pavia, Italy; l.caliogna@smatteo.pv.it

**Keywords:** hemophilia, platelet, joint, synovitis

## Abstract

Intra-articular blood, iron and hemosiderin, hydroxyl radical cytokines, and neo-angiogenesis cause synovial inflammation, which leads to cartilage and joint damage. Platelet-rich plasma (PRP) inhibits most of the mediators that produce and maintain synovitis. We compile here our work showing the clinical effectiveness of intra-articular PRP injections and their potential role in stopping articular cartilage damage due to bleeding and its possible repair. A total of 116 joints, including knees (63%), elbows (19.8%), and ankles (17.2%), were treated with intra-articular injections of PRP. Moreover, we also show here the number of extracellular DNA traps (ETs) and the PRP effect in the synovial fluid of patients at the time of treatment and six months after. Clinically, it is demonstrated that PRP is effective in reducing bleeding episodes (*p* < 0.001) and pain (*p* < 0.0001) and improving the hemophilia joint health score (HJHS) (*p* < 0.001) at one year of follow-up. Furthermore, our results demonstrate that PRP inhibits ET formation *in vitro* and reconstitutes the immune system’s cellular components in the synovial fluid of patients after treatment. We conclude that PRP can be considered an effective, safe, and easy treatment for hemophilic synovitis.

## 1. Introduction

Post-bleeding synovitis in patients with hemophilia is a devastating pathology that generates joint destruction, pain, loss of joint range of motion, and depression and alters the patient’s relationships and social life. The World Federation of Hemophilia defines chronic synovitis as synovial inflammation persisting over and beyond three months. The development of synovitis is the first step towards the development of chronic arthropathy and joint destruction that includes different pathophysiological mechanisms [1]. Studies have shown that exposure to blood *in vitro* produces chondrocyte apoptosis and affects the renewal of the cartilage matrix exposed to whole blood [2]. Synovial proliferation is stimulated by different pathophysiological mechanisms [3,4]. Hemosiderin is phagocytized by the synoviocytes, which act as macrophages, and iron is deposited in the synovial tissue, generating synovial proliferation [5]. Mononuclear cells produce cytokines such as interleukins (IL-1ß) and tumor necrosis factor (TNF), which generate several other proinflammatory cytokines, such as IL-6 and IL-8, and also cause the synthesis of proteases including matrix metalloproteinase [6,7,8]. IL-1ß stimulates the production of hydrogen peroxide (H_2_O_2_) by chondrocytes. H_2_O_2_ reacts with iron derived from erythrocytes, through the Fenton reaction, and forms hydroxyl radicals, resulting in the apoptosis of chondrocytes, which causes the loss of proteoglycans; this decreases the mechanical properties of the cartilage [9]. If the regional harmful stimulus that produces synovitis is of sufficient magnitude, it can cause the regional acceleratory phenomenon (RAP), depending on the magnitude of the stimulus and the response of the individual [10]. 

On radiography and magnetic resonance imaging, it is expressed as regional osteoporosis. Local hypervascularization generates hyperactivity of the growth plate, leading to the accelerated and desynchronized regional growth of the affected limb, which gives rise to deformities; this will facilitate the appearance of future arthropathy [11].

Synovial hypoxia is caused by the growth of the synovium, inflammation, and reduced blood flow upon increased intra-articular pressure [12]. This phenomenon triggers the release of the pro-angiogenic mediator’s vascular endothelial growth factor (VEGF), matrix metalloproteinase-9 (MMP-9), and stromal cell-derived factor-1 (SDF-1) [13,14]. Synovial hyperplasia leads to the formation of new blood vessels, which makes the synovial tissue more susceptible to intra-articular bleeding, facilitating new recurrent bleeding and maintaining the synovitis production mechanism. Platelet-rich plasma (PRP) has inhibitory effects on several of these pathophysiological mechanisms of chronic synovitis in patients with hemophilia [15,16]. The therapeutic mechanisms underlying PRP in the pathophysiological mechanisms of damage in chronic hemophilic synovitis (CHS) have not yet been clearly understood. In addition, PRP has tissue-regenerative effects carried out by platelet derivatives, which potentially allow the regeneration of cartilage after damage [17]. Alpha granules release growth factors and also contribute cytokines, chemokines, and proteins involved in chemotaxis, cell proliferation, and maturation and in controlling inflammatory processes. In addition to alpha granules, platelets contain dense granules with ADP, ATP, calcium ions, histamine, serotonin, and dopamine, which also contribute to tissue regeneration. Platelets also contain lysosomal granules. These granules secrete molecules such as acid hydrolases, cathepsin D and E, elastases, and lysozyme [18]. 

As explained in the previous paragraphs, intra-articular PRP is the only existing method for the treatment of CHS; it generates a pathophysiological blockage of the biological cascade of damage and also has the potential ability to repair damaged cartilage.

The aim of this work is to demonstrate the effectiveness of intra-articular PRP injections to block the pathophysiological cascade of articular cartilage damage due to bleeding and its possible repair through these autologous biological properties.

## 2. Results

### 2.1. Clinical Study

Eighty-four patients with 116 chronic hemophilic synovitis joints were treated: 40 patients had only one affected joint, twenty-two had two affected joints, twenty patients had three affected joints, and two patients had four simultaneously affected joints. The incidence of the affected joints per patient was 1.38.

**Figure 1:**
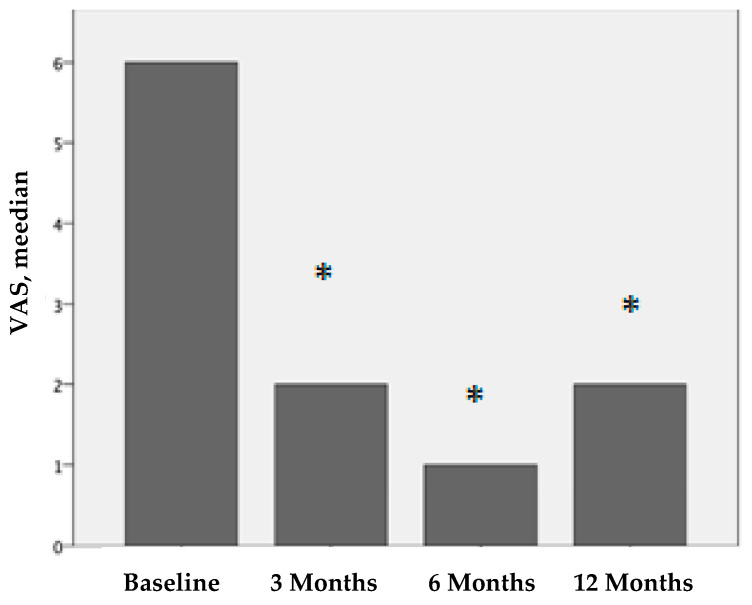
VAS values before treatment and 3, 6, and 12 months after PRP treatment. We compare the results before treatment vs. 3 months after treatment, before treatment vs. 6 months after treatment, and before treatment vs. twelve months after treatment. The results are statistically significant (* *p* < 0.001).

**Figure 2:**
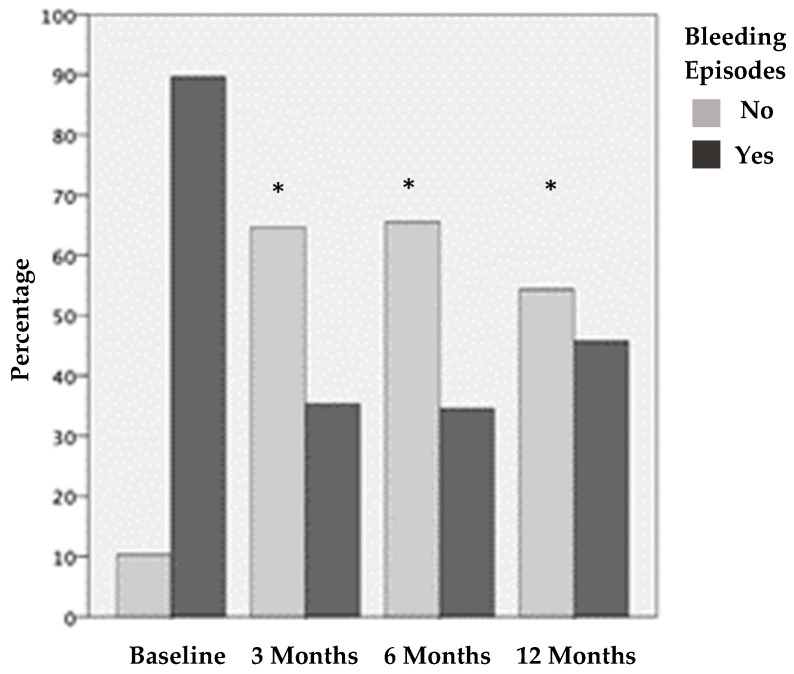
Joints with and without bleeding episodes before treatment and 3, 6, and 12 months after PRP treatment. The results show statistically significant differences before vs. the three different post-treatment periods evaluated (* *p* < 0.001).

**Figure 3:**
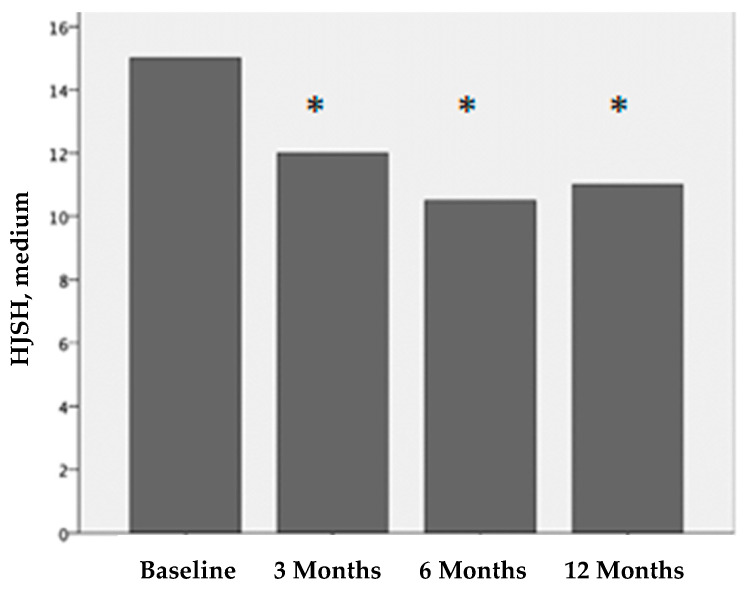
Hemophilia joint health score (HJHS) before treatment and 3, 6, and 12 months after PRP treatment. The differences before and after treatment are statistically significant (*p < 0.001).

Figure 1, Figure 2 and Figure 3 show the clinical evaluations of the 116 joints before treatment and 3, 6, and 12 months after treatment, in terms of the visual analog scale (VAS), bleeding episodes (BE), and hemophilia joint health score (HJHS).

All patients reported pain relief after PRP treatment. The VAS scores for pain perception were statistically significant. The median VAS score before treatment was 6 (IQR 4-7), that 3 months after treatment was 2 (IQR 0-4), that 6 months after treatment was 1 (IQR 0-4), and that 12 months after treatment was 2 (IQR 1-6). These differences were statistically significant (*p* < 0.001).

Before treatment, 104 (89.7%) joints presented BE in the last month. This was reduced by 54% at three months after treatment; only 35.3% of the joints showed BE in the last month. After 6 months, 34.6% of the joints had BE in the last month, and, after 12 months, 45.7% of the joints had BE in the last month. The decrease in the joint BE was statistically significant (*p* < 0.001) according to the frequency of joint bleeding episodes before treatment and after 3, 6, and 12 months of treatment. There were no statistical differences at 3 vs. 6 months after treatment and 6 vs. 12 months after treatment.

Before treatment, the median HJHS for each affected joint was 15 (IQR 11-17). Three months after treatment, it was 12 (IQR 10-14); six months after treatment, it was 10.5 (IQR 8-13); and twelve months after treatment, it was 11 (IQR 8-13). The differences in the HJHS scores before treatment vs. 3 months after treatment, vs. 6 months after treatment, and vs. 12 months after treatment were statistically significant (*p* < 0.001). There were no statistical differences at 3 vs. 6 months after treatment and 6 vs. 12 months after treatment.

### 2.2. Experimental In Vitro Study

#### 2.2.1. ET Analysis of Synovial Fluid of Patients with CHS

**Figure 4:**
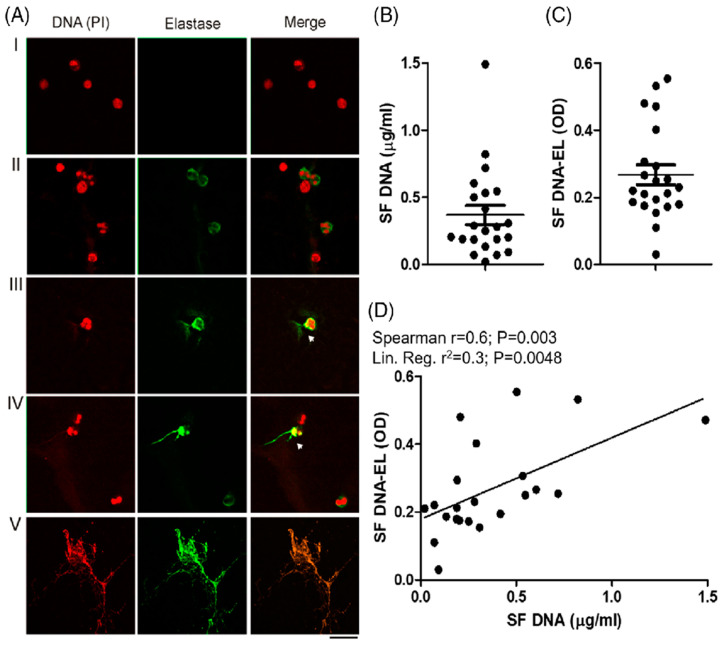
Presence of ETs in synovial fluid of patients with CHS. (**A**) The presence of ETs in 22 SF samples was analyzed after being stained with propidium iodide (DNA is colored in red) and anti-human elastase (elastase is colored in green) using fluorescence confocal microscopy (60X). Representative images of attached cells stained with irrelevant antibody isotype or PMA-stimulated neutrophils were used as negative (**I**) or positive ET release control (**II**) signals. The incipient release of DNA strands (red) with bound elastase (green) is indicated with white arrows in panels (**III**,**IV**). Massive and complete DNA release is shown in panel (**V**). (**B**) Quantification of cell-free DNA and (**C**) DNA-EL complex measured in the 22 SF samples. (**D**) Statistical associations between these last two parameters were analyzed by Spearman’s correlation and linear regression models. As presented in Oneto et al. [19].

The presence of extracellular DNA traps (ETs) in the synovial cavities of patients with CHS is shown in Figure 4A–C, and Oneto et al. [19] suggest ETs as potential biomarkers of CHS. A strong and significant positive correlation was also observed between the synovial levels of DNA and those of DNA elastase (Spearman r = 0.6; *p* = 0.003), indicating that the presence of synovial DNA is partly derived from ETs (Figure 4D). Furthermore, we showed in Oneto et al. [19] that the positive correlations between the worse clinical parameters of patients with CHS (BE/HJHS) and the presence of DNA and DNA elastase suggest that synovial ETs could be a good biomarker of articular pathologies. In the same sense, the systemic level of ETs was also significantly increased in the plasma of patients with CHS, as shown in Oneto et al. [19].

**Figure 5:**
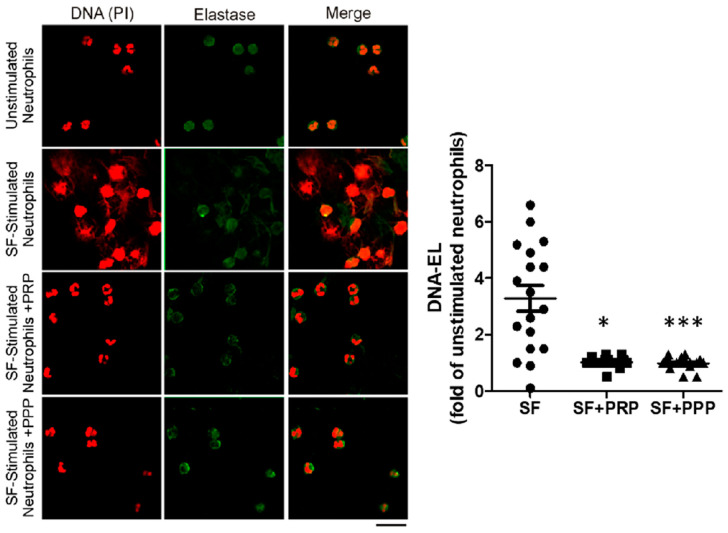
The synovial fluid of patients with CHS contains soluble ET inducer factors that are inhibited by plasma. *In vitro* ET induction was evaluated by stimulating healthy neutrophils with 10% of SF for 2 h, in the presence or absence of PRP or PPP. Neutrophils incubated with 10% of medium without SF were used as controls for unstimulated neutrophils. After fixation, cells were stained with PI (red) and anti-human elastase (green). Representative fluorescence confocal microscopy images (60X) of NETs induced by SF samples (N = 18). DNA-EL levels were quantified in supernatants by ELISA and expressed as fold changes of unstimulated neutrophils (*n* = 18, * *p* < 0.05, *** *p* < 0.001 vs. SF-stimulated neutrophils (SF) without PRP/PPP, Kruskal–Wallis test). As presented in Oneto et al. [19].

Interestingly, *in vitro* neutrophils ETs (NETs) induced by SF and measured by the DNA elastase levels were reduced when treated with PPP or PRP, as shown in Figure 5 and Oneto et al. [19]. These results demonstrate the protective effect of soluble plasma factors against pathogenic ETs. Furthermore, we also reported a significant reduction in ETs associated with clinical improvements in four patients after 1 month of PRP treatment, reaching no BE and a reduction in the HJHS in three of them; see Oneto et al. [19]. Our data suggest that a possible mechanism underlying the therapeutic effect of the intra-articular injection of PRP is the reduction of ETs.

#### 2.2.2. Hemoglobin Oxidation Is Inhibited by Plasma Contained in PRP

**Figure 6:**
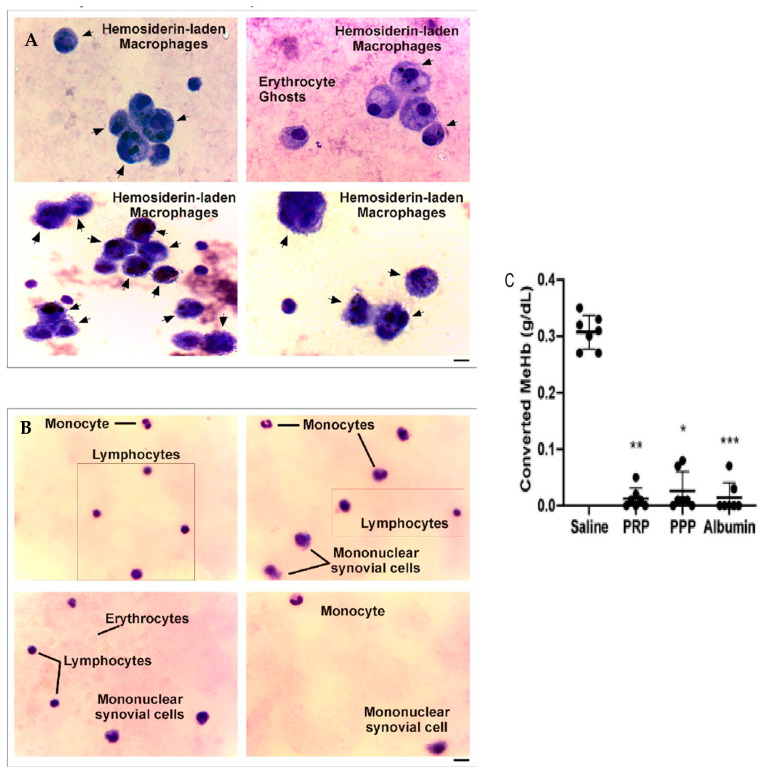
Synovial fluid analysis before and after PRP treatment of patients with CHS. (**A**,**B**) Synovial fluid samples were spread on glass coverslips, fixed, and stained with Giemsa. Images were obtained by optical microscopy in different fields of slides and represent the cellular composition (black arrows) of synovial fluid from the same patient before and after 6 months of PRP treatment (magnification 400X, scale bar 20 μm). (**C**) Converted MeHb (g/dL) was calculated based on the concentration of stock buffered Hb solution quantified using a hematology analyzer (*n* = 7, * *p* < 0.05, ** *p* < 0.01, *** *p* < 0.001 vs. saline). As shown in Caviglia et al. [20].

The analysis of microscopy pictures of synovial fluid samples from patients before PRP (Figure 6A) and dyed with Giemsa showed the presence of macrophages with cytoplasmic hemosiderin deposits stained as dark violet–blue dots. In contrast, at 6 months after one dose of an intra-articular injection of PRP (Figure 6B), the samples present almost a normal cellular composition characterized by constitutive populations (Figure 6), complemented by a clinical improvement in joint inflammation and crackles and an upgrade in muscle strength that resulted in a reduction in the HJHS, joint pain (VAS), and bleeding episodes.

When analyzing the levels of oxidized hemoglobin (Hb) and methemoglobin (MeHb), we found the presence of this compound before PRP treatment, in comparison to the undetected levels after treatment with PRP. Moreover, when evaluating the oxidation of Hb to MeHb (hem-Fe^2+^ in hem-Fe^3+^) induced by the addition of K_3_FeCN_6_ in the presence of PRP, this phenomenon was completely inhibited in the presence of PRP, PPP, and purified serum albumin, whereas, in the control condition (saline), the 100% Hb stock solution (0.31 ± 0.01 g/dL) was converted to MeHb (Figure 6C) [20].

These results suggest that the anti-oxidative effect of plasma on Hb observed *in vitro* was also reflected in the synovia of CHS patients, preventing, in this way, the accumulation of toxic intermediate forms of free iron.

## 3. Discussion

Platelet-rich plasma obtained from patients’ own blood is safe, low-cost, and has few adverse effects [21]. The simplicity and safety of the procedure allow it to be used worldwide. All parameters evaluated remained below the pre-treatment levels, but they were higher at 12 months than at 6 months. We believe that repeating the injections once a year could protect the joints [22]. This *in vitro* study demonstrates that the synovial fluid is bloody in CHS and the cellular composition presents hemosiderin-laden macrophages and ghost erythrocytes. After PRP treatment, the synovial fluid had a light yellowish appearance and its cellular composition contained monocytes, lymphocytes, and mononuclear synovial cells. This new composition of the synovial fluid can fulfill the function of nourishing the cartilage cells [23]. This study of synovial fluid in patients with CHS suggests, for the first time, that ETs are a potential biomarker and a new therapeutic target for CHS. The diminution of ETs in the presence of PRP, observed *in vitro* as well as in patients’ fluid samples after treatment with PRP, suggests that the dampening of ETs is a possible mechanism underlying the therapeutic intra-articular injection of PRP in CHS [19]. The inhibition of the proinflammatory cytokines produced by PRP has been demonstrated by several authors [24,25]. In the same way, many other authors have demonstrated the blocking of the action of TNF [26,27]; moreover, angiogenesis was promoted [28,29] and the deregulated overexpression of c-Myc and Mdm2 was observed [30,31]. The only pathophysiological mechanism of damage in CHS, for which the action of PRP was unknown, was iron oxidation, produced by the Fenton reaction. The mechanism of the inhibition of iron oxidation by PRP was shown, suggesting that the anti-oxidative effect of PRP is due to the presence of plasma proteins, probably attributable to albumin [20]. Consequently, the only pathophysiological mechanism of joint damage in CHS that is not blocked by PRP is chondrocyte apoptosis, secondary to the exposure of the joint surface to blood due to hemarthrosis [2]. For all previous exposures, we consider that treatment with PRP, which inhibits different pathophysiological mechanisms, could be considered a biological synovectomy. Open surgical synovectomy for the treatment of hemophilic synovitis was first described by Storti et al. [32]. The invasiveness of the procedure and the loss of movement made open synovectomy an undesirable procedure. Other treatments, such as radioactive synoviorthesis, are expensive and require several applications. The two most used radiopharmaceuticals are yttrium citrate (90Y) and rhenium sulfide (186Re), which release high-energy beta particles to the innermost cell layer of the synovial membrane, causing pronounced cell death, the obliteration of the capillary blood supply, and therefore fibrosis and sclerosis of the synovial membrane [33]. Radioactive substances have a penetration depth of 1.2 mm for rhenium and 3.6 mm for yttrium, but the articular cartilage is also exposed to colloidal beta-emitting radionuclides, and these can be potentially harmful to cartilage cells, and we are not able to differentiate between healthy cells and those affected by joint bleeding. Chemical synoviorthesis via intra-articular application has shown good results in small joints (elbow and ankle) but not in large ones, such as the knee [34]. Synovioangiolysis, which is another treatment method, is expensive, requires trained radiologists to perform it, and cannot be performed on the ankle joint due to the risk of distal ischemia [35]. The advantage of PRP treatment in patients with CHS is that it only acts on tissue affected by intra-articular bleeding and it provides the possibility of the repair of the affected cartilage. Xu et al. have shown that PRP can reduce the activation of NF-κB induced by IL-1β, thus inhibiting the inflammatory process of chondrocytes [36]. Chondro-protection can occur by increasing anabolic factors such as TGF-β1 and reducing MMPs to limit cartilage degradation [37,38]. Finally, different studies have shown that PRP has the ability to promote the chondrogenic differentiation of stem cells [39,40]. 

The limitations of our studies are the lack of ultrasonography parameters to evaluate the degree of synovitis after the treatment with PRP, the lack of analysis of the synovial fluid of a greater number of patients, and the short follow-up of this group of patients.

In conclusion, and despite some limitations, the clinical and laboratory studies compiled and summarized in this paper demonstrate that intra-articular PRP is effective in controlling intra-articular bleeding, relieving pain, and improving HJHS scores. We also demonstrate that ETs are present in the SF of patients with CHS and correlate with joint damage and functional alterations, determined as increased BEs and worse HJHS scores. Interestingly, the diminution of ETs in the presence of PRP, the inhibition of the Fenton reaction, and the normalization of the joint cellular composition observed *in vitro*, as well as in patients’ fluid samples after PRP treatment, mechanistically support the notion that PRP can be considered as a therapeutic treatment for CHS. Finally, PRP is an effective, safe, and easy treatment for hemophilic synovitis, being accessible anywhere in the world. We consider that biological synovectomy is simple to perform, non-painful, safe, and of low cost, and it also has a potential reparative effect on cartilage, a property that none of the other treatments provides.

## 4. Materials and Methods

### 4.1. Study Population

From 2017 to 2022, 84 patients with 116 chronic hemophilic synovitis joints were recruited for the present study. The patients’ mean age was 24.5 years old (8–60); 75 (89.3%) patients had hemophilia type A and nine (10.7%) patients had hemophilia type B; 74 (88%) patients had severe hemophilia and ten (12%) patients had moderate hemophilia. At the first meeting, clinical evaluations of the bleeding episodes (number of bleeding episodes in the last month), the hemophilia joint health score for each affected joint, and the pain perception (VAS) before (the same day of the procedure) PRP treatment were performed. All patients recruited in this study (84 patients) accepted the treatment with PRP and no patient was excluded from the study.

Follow-ups were carried out at three, six, and twelve months after treatment for clinical evaluations of BE, HJHS, and VAS, performed by the same professional specialist in hemophilia.

For the analysis of the cellular composition of the synovial fluid, in a small group of 21 patients, we collected SF after PRP injection (it was collected from 22 joints: one ankle and 21 knees, where one was affected bilaterally). These patients were selected because they presented a swollen joint with excess synovial fluid. Nineteen patients had hemophilia type A (17 severe, 2 moderate) and two had type B, both severe. The mean age was 28 years old (13–60). We also performed a follow-up after PRP treatment and the aspiration of SF was performed just before and at 2 weeks after the application of the intra-articular injection of PRP.

- Inclusion Criteria: Patients with chronic synovitis diagnosed by clinical exam, ultrasound, and/or MRI.
- Exclusion Criteria: Patients with grade 5 arthropathy (osseous ankylosis) according to Arnold and Hilgartner’s radiological classification and clinical exam. Patients with loss of skin or active infection in the joint were also excluded.

#### Platelet-Rich Plasma Procedure

The affected joints were treated with fresh PRP. After blood extraction, patients were given the corresponding coagulation factors indicated by the treating hematologist. The recommendation was to infuse factor VIII or IX, as appropriate, 10 min before PRP injection, to reach clotting factor activity of 50% and to maintain factor levels ≥30% for the next three days.

In brief, PRP was prepared as follows: blood was extracted with the BD Vacutainer^®^ (Becton Dickinson, Franklin Lakes, NJ, USA) collection set (butterfly needle, holder, and 8.5 mL ACD tubes). We used a single-speed method for PRP preparation, consisting of 8 min centrifugation at 1800 rpm or 360× *g* (Presvac, Buenos Aires, Argentina) at room temperature. Then, the PRP fraction was carefully harvested with a pipette (in order to minimize white or red blood cell aspiration) and placed in a sterile syringe. This procedure was performed in a Class II biosafety cabinet (Biobase, Jinan, China).

The intra-articular PRP injections were performed under sterile conditions in the operating room. The average delay between blood extraction and PRP injection was one hour. The mean volume of extracted blood for PRP preparation was 15 mL (12–24 mL). This volume depended on the number of joints to be treated simultaneously. The mean volume of injected PRP was 4 mL (2–5 mL) for each affected joint and varied according to the size of the joint treated.

### 4.2. In Vitro Study

#### 4.2.1. Chronic Hemophilic Synovitis Evaluation and Synovial Fluid Analysis

Ultrasound and/or magnetic resonance imaging was performed for the diagnosis of all chronic synovitis joints. For the quantification of the levels of extracellular DNA traps (DNA/DNA elastase), synovial fluid samples from CHS patients before and after PRP treatment were centrifuged (1100× *g*, 10 min) to obtain cell-free supernatants. The quantification of the ETs in the supernatants was performed by PicoGreen fluorescence (DNA) and ELISA (DNA elastase), and microscopy pictures were analyzed by confocal fluorescence microscopy using an FV-1000 microscope (Olympus, Tokyo, Japan), as described in Oneto et al. [19].

#### 4.2.2. Study of Hemoglobin Oxidation Induced *In Vitro* in the Presence of Plasma

The analysis of the cellular composition was performed by spreading one drop of this fluid on a glass coverslip, after which it was fixed and stained with Giemsa. The oxidation of Hb to methemoglobin through the Fenton reaction was induced in the presence or absence of peripheral blood-derived platelet-rich or platelet-poor plasma (PRP/PPP) or albumin [41]. The relevance of the *in vitro* findings was analyzed in synovial fluid (SF) samples from patients with chronic synovitis obtained before and after 6 months of PRP intra-articular injection. The determination of MeHb in the synovial fluid before and after PRP treatment was obtained by diluting synovial fluid 1:10 in Sorenson’s buffer (0.066 M, pH 6.8), and the absorbance (630 nm) was determined using a 96-well plate reader. These procedures were performed as described in Caviglia et al. [20].

### 4.3. Statistical Analysis

We performed an experimental study to analyze the variables (BE, VAS, and HJHS), before and after PRP intra-articular injection during a twelve-month follow-up. The median and interquartile range (IQR) were reported. To determine the distribution symmetry, the Shapiro–Wilk or Kolmogorov–Smirnov tests were used, as appropriate. Categorical variables were reported as absolute numbers and percentages. For categorical variables, chi-square or Fisher’s exact tests were used. For continuous variables, Student’s *t*-test or the Mann–Whitney test was performed. Data obtained three, six, and twelve months after treatment were independently analyzed. 

The Shapiro–Wilk test was used to define normality and equal variance. If the normality assumption was not fulfilled, non-parametric tests were performed. The association between the experimental and clinical parameters was analyzed following models of Spearman or Pearson correlations and linear regression. 

A *p*-value lower than 0.05 was considered statistically significant. All statistical analyses were performed using the SPSS Statistics v. 24 software (SPSS Inc., Chicago, IL, USA).

## Data Availability

Data are contained within the article.
